# Disclosure of Intimate Partner Violence and Associated Factors among Victimized Women, Ethiopia, 2018: A Community-Based Study

**DOI:** 10.1155/2020/6513246

**Published:** 2020-07-21

**Authors:** Liyew Agenagnew, Bosena Tebeje, Ruth Tilahun

**Affiliations:** ^1^Department of Psychiatry, Faculty of Medical Sciences, Institute of Health, Jimma University, Jimma, Ethiopia; ^2^School of Nursing, Faculty of Health Sciences, Institute of Health, Jimma University, Jimma, Ethiopia; ^3^Department of Midwifery, College of Health Sciences and Medicine, Dilla University, Dilla, Ethiopia

## Abstract

**Background:**

Disclosure is a vital step in the process of finding a lasting solution and breaking the abuse chain in a victim woman by the intimate partner.

**Objectives:**

This study is aimed at assessing the disclosure of intimate partner violence and associated factors among victim women in Dilla town, Gedeo Zone, South Ethiopia, 2018.

**Methods:**

A community-based cross-sectional study design triangulated with the qualitative method was employed. Data were collected from 280 women victims of intimate partner violence using pretested, structured, and interviewer-administered questionnaires. SPSS version 20.0 software was used for analysis. Binary logistic regression and a multivariate logistic regression model were fitted to assess the association between the independent and dependent variables. Qualitative data were collected through in-depth interviews and categorized into themes and triangulated with the quantitative result.

**Results:**

Half of the respondents (51%) disclosed intimate partner violence. Partner alcohol use (AOR = 1.99; 95% CI:1.18, 3.34), women experiencing a single type of intimate partner violence (AOR = 0.38, 95% CI: 0.17, 0.79), women having strong social support (AOR = 2.52; 95% CI:1.44, 4.41), and women whose partners' having primary (AOR = 2.04; 95% CI:1.07, 3.9) and secondary education (AOR = 2.16; 95% CI: 1.07, 4.33) were significantly associated with the disclosure of intimate partner violence as the qualitative result shows most of the women prefer their family to disclose and those who kept silent were due to economic dependency, societal norms towards wife beating, arranged marriage, and not getting the chance especially those who went to the hospital.

**Conclusion:**

Nearly 50% of victims of intimate partner violence women disclose intimate partner violence to others. Thus, it is needed for stakeholders to use their efforts to further increase the disclosure of violence and respect women's rights and equality.

## 1. Introduction

Intimate partner violence is fundamental to public health challenges and violates women's human rights. It is one of the most common forms of violence in the direction of women and includes bodily, sexual, and emotional abuse and controlling behaviors by the intimate partner [1].

Approximately 1 in 3 women worldwide have experienced either bodily and/or sexual intimate partner violence in their lifetime [1, 2].

Most victims of intimate partner, women, keep silent, and only a few women disclosed violence to others, in the world only 20% [3], in America, more than 74% intimate partner violence survivor women stayed with an abuser and does not disclose intimate partner violence [4], 77.9% of Serbian women did not disclose intimate partner violence [5], in Nigeria, only 46% women disclosed intimate partner violence to formal or informal services [6], and in Tanzania, 41% of women do not disclose intimate partner violence [7–9].

Not disclosing intimate partner violence may lead to a host of negative sexual and reproductive health consequences for women, including unintended and unwanted pregnancy, abortion and unsafe abortion, sexually transmitted infections including HIV, pregnancy complications, pelvic inflammatory disease, urinary tract infections and sexual dysfunction, physical and mental injury, and death [10–12].

Most frequently mentioned factors in previous studies as the reason behind not disclosing intimate partner violence to others were economic and sociopolitical discrimination of women, being dependent on partners' income [4, 13], the severity of the violence [14–16], those who are severely violated more likely to disclose their experience to the formal social services [17], stress, accepting violence as normal [14], concerns about bringing bad name to the family, lack of confidence, shame/embarrassment/fear of getting blamed, fear for children's future life, women who were unemployed and pregnant, and lack of encouragement by family members [15, 16, 18–20] and educational status [21, 22].

In Ethiopia, intimate partner violence accounts in one-third of the women [23]; however, victimized (violated) women's disclosure status and factors associated with it were not investigated explicitly especially in women found in a rural area. So this study attempted to assess the magnitude of disclosure of intimate partner violence and factors associated with it, in the rural community of Ethiopia, from March 25 to April 25, 2018, by using both qualitative and quantitative methods, which might be helpful health care providers to screen women for intimate partner violence and aid to disclose it as well it will be helpful for different stakeholders to address and intervene the reasons for not disclosing violence to others despite the fact that they are suffering from it.

## 2. Methods and Materials

### 2.1. Study Area and Period

The study was conducted from March 25 to April 25, 2018, in Dilla town administrative center of the Gedeo Zone in the Southern Nation Nationalities and People Region, which is found 360 km away from Addis Ababa and 90 km from the capital city of the region Hawassa. The total area of the town is 135 km^2^. Total population is 102,624, among which 50,286 (48.9%) are males and 52,338 (51.1%) are females. The total reproductive age group women are 20,204. The total number of households is 20,944. The dominant ethnic group is Gedeo, and most of the people speak Gedeo-Offa and Amharic languages.

### 2.2. Study Design

A community-based cross-sectional study design was employed.

### 2.3. Populations

#### 2.3.1. Source Population

The source population was reproductive age women with the experience of intimate partner violence and residing in Dilla town.

#### 2.3.2. Study Population

The study population was reproductive age women with the experience of intimate partner violence in the selected kebele households (Haroresa, Odaya, and Harsu kebeles) in Dilla town.

### 2.4. Inclusion Criteria and Exclusion Criteria

#### 2.4.1. Inclusion Criteria

Women whose ages are between 18 and 49 years, have/had an intimate partner, and had intimate partner violence before the data collection period were included in the study.

#### 2.4.2. Exclusion Criteria

Severely ill women that are not able to give information at the time of data collection were excluded from the study.

### 2.5. Sample Size Determination

The sample size was determined by using a single population proportion formula by considering the following assumptions: *P* = 31%—prevalence of women disclosing violent experience in North West Ethiopia [7], *d* = margin of error of 0.05 with 95% confidence interval, and *α* = 0.05 (level of significance) using the formula *n* = (Z*α*/2)^2^ *p* (1 − *p*)/*d*^2^; then the calculated sample size became 329, Since the source population is below 10,000, correction formulae were used nf = *n*/1 + *n*/*N*, where *n* = a minimum sample size, *N* = total number of women that experienced intimate partner violence in the study area (1958), and NF = minimum final sample size; it becomes 282. By adding 10% nonresponse rate, the final total sample size was 310.

For the qualitative part, the sample size was determined by saturation of the information.

### 2.6. Sampling Technique

For the quantitative part, from the total of nine kebeles found in Dilla town, three kebeles called Haroresa kebele (located in Harowelabu subcity), Odaya kebele (located in Sesa subcity), and Harsu kebele (located in Bedecha subcity) were selected by using the lottery method. In those selected kebeles, there were a total number of 6117 households, i.e., 3295, 1409, and 1413, respectively. We screened women found in those kebeles (10,235) for having intimate partner violence, and we got a total of 1958 women who had a positive reply for intimate partner violence from three kebeles. Then, by using a simple random sampling technique, we selected our study participants by the lottery method from the prepared sampling frame. Data collectors trace the selected participants for interviews with the help of health extension workers who know the kebeles very well using the coded household and women selected from the sampling frame. One house was visited with a maximum of three times, but if data collectors cannot get respondents for the third time, they considered it nonrespondent.

For the qualitative part, a purposeful sampling technique was used. Potential respondents were selected for the in-depth interview. Key informants were selected based on their experience of violence in their lifetime and who can express the required information about the problem clearly. A total of 8 women participated in this study.

### 2.7. Data Collection Procedures and Instruments

Data was collected through face to face interviews moving from house to house by using 6 female diploma nurses as a data collector after written consent was obtained from the respondents. The data collection process was conducted individually at a convenient location for the respondents.

To assess disclosure of intimate partner violence, a validated tool with 13 items was adapted and used by reviewing different literatures [24, 25]; for other variables, structured questionnaires were used, which were prepared in the English language then translated to the local languages, *Amharic* and *Gedeo-Offa* for data collection purpose. The questionnaires have five parts, involving the sociodemographic and behavioral characteristics (16 items), reproductive health-related (10 items), social support (3 items), and women empowerment (9 items).

Intimate partner violence was assessed by the self-reported experience of one or more acts of any form of violence by a current or former partner including physical, sexual, psychological, and controlling behavior during the last 12 months.

A woman who shares her violent experience by their former or current intimate partner to any other person or any service was taken as having intimate partner violence disclosure.

Test-retest reliability was measured to determine the reliability of the tool, and to measure its internal consistency, Cronbach's alpha was used and it becomes 0.7.

For the qualitative part, semistructured guiding questions with five items were used and data were collected by the principal investigator. The average length of the interview was 30 minutes. The key guiding questions were as follows: (1) Do you know what intimate partner violence is? (2) What are the different types of intimate partner violence? (3) Does anyone of your neighbor or relative share their violent experience to you? (4) Have you ever share your intimate partner violence experience to others? (5) Why you share your violent experience with others? Audiotape recorders and notes were used to document the data at the time of in-depth interviews and key informant discussion.

### 2.8. Data Quality Control

To assure the data quality, one-day training was given for data collectors and supervision was made at the time of data collection in each kebele; also, data collection tools were translated from English to Amharic and Gedeo-Offa by experts and back-translated to English to check the consistency. Moreover, a pretest was done in Boiti kebele by taking 5% [16] of the total sample size before one week of the actual data collection, and data were checked for completeness and corrective measures were taken immediately.

### 2.9. Data Processing and Analysis

Data were cleaned and entered using Epi data version 3.1 then exported to SPSS version 20 for analysis. Data were analyzed using SPSS version 20. Descriptive statistics were computed to determine the prevalence of disclosure of intimate partner violence. Binary and multivariate logistic regression analysis was done to see the association between the dependent (disclosure status of intimate partner violence) and independent variables. Binary logistic regression was used to identify variables that are a candidate for multivariate logistic regression analysis at *p* value < 0.25, and multivariate logistic regression analysis was used to determine the factors that are independently associated with disclosure status of intimate partner violence at *p* value < 0.05 with a 95% confidence level. Finally, variables with *P* value < 0.05 were considered statistically significant. Model fitness was assessed through the Hosmer and Lemeshow test (*p* = 0.087).

For the qualitative part, the data was transcribed and translated from Amharic to the English language by the research team and language teachers independently. Texts were thoroughly read repeatedly to identify thematic areas. Their inductive meanings were extracted and described in narratives thematically. Three of the authors have participated in the thematic analysis. Finally, ideas were triangulated with the quantitative result.

### 2.10. Ethical Considerations

Ethical clearance was obtained from Jimma University Institute of Health Institutional Review Board with approval number IHRPGD/69/19. Written consent was obtained from the study participants, and the data were collected at a private place to assure confidentiality of the participants. Participants have the right to stop the interviews at any time if they were not interested. Participant information was locked in the file cabinet in the personal possession of the researcher only and will be burned after 5 years. For women who experienced intimate partner violence at the time of screening, the linkage was established with the women's affairs office depending on their willingness for further discussion about violence and to find a solution.

## 3. Results

### 3.1. Sociodemographic Characteristics of Study Participants

Two hundred eighty victimized women have participated in the quantitative study that yields a response rate of 90%. The mean age of women was 30.4 (SD ± 5.7) years. The majority (41.6%) of women were Gedeo by their ethnicity and Protestant followers by their religion (43.9%) and 88 (31.1%) were housewives, and about 207 (74%) household heads were partners/husbands; 57 (20.4%) of women reported that their partner had other relationships with other women; majority of the participants (183 (65.4%)) reported that their partner drinks alcohol (Table 1).

### 3.2. Types of Intimate Partner Violence and Social Support

The most common type of intimate partner violence reported was controlling behavior (236 (84.3%)) followed by physical and emotional violence accounted for 209 (74.6%) and 183 (65.3%), respectively, while sexual violence was the least reported (106 (38.9%)) type of intimate partner violence (Table 2).

More than half (239 (85.4%)) of victimized women had strong social support, and the other (41 (14.6%)) had poor and moderate social support.

### 3.3. Women Empowerment

From the total of 280 victimized women, 107 (62.5%) of them had access to information about intimate partner violence, 193 (68.9%) were able to visit their family, 244 (87.1%) and 223(79.6%) could independently make a decision on their own and children's health, respectively, and more than half of respondents (166 (59%)) could contribute and make the decision on household purchases.

### 3.4. Reproductive Health-Related Characteristics

From the total of 280 victimized women, majority (171 (61.1%), 151(53.9%)) got married and had their first sexual intercourse after the age of 18, majority (182 (65%)) had 1-4 children, and more than one-third (104 (37.1%)) of women were pregnant during the last 12 months.

### 3.5. Disclosure of Intimate Partner Violence

More than half of victimized women (144 (51.4%)) disclosed their experience of violence by their intimate partner to someone (local elders, family, police, and religious fathers), and most of them (107 (73.8%)) disclosed their intimate partner violence experience to their family (Table 2).

**Figure 1:**
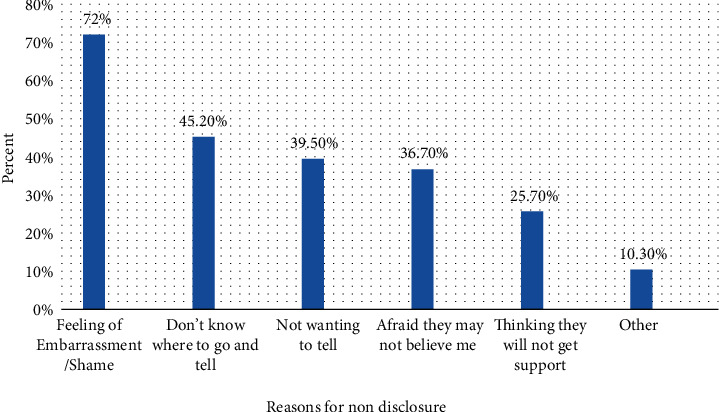
Reasons for not disclosing intimate partner violence among violated women in Dilla town, Ethiopia (*n* = 136). ^∗^Other—fear of revenge: 10 (7.3%); warned not to tell to any one: 4 (3%).

From the total victimized women, 136 (48.6%) do not disclose their violent experience by their intimate partner to others due to the following reasons: feeling of embarrassment/shame (72%), do not know where to go (45.2%), not wanting others to be involved (39.5%), and afraid they may not believe me (36.7%) (Figure 1).

### 3.6. Factors Associated with Disclosure of Intimate Partner Violence

All variables were assessed independently with the dependent variable in the bivariate logistic regression analysis, and those variables having a *p* value < 0.25 were candidates for multivariate logistic regression analysis (Table 3). Then, from the multivariate logistic regression analysis at *p* value < 0.05, partner alcohol use (*p* value = 0.012), partners' educational status (*p* value = 0.014), social support (*p* < 0.01), the total number of intimate partner violence experienced (*p* value = 0.003) were significantly associated with disclosure of intimate partner violence.

The Hosmer and Lemeshow goodness of fit test (*p* = 0.087) provides evidence of model fitness with the predicators.

Women whose partners drink alcohol are nearly 2 times more likely to disclose their experience to others (AOR = 1.99, 95% CI: 1.18, 3.34) as compared to those whose partners do not drink alcohol.

Women who experienced only one type of violence were 62% less likely to disclose their violent experience compared to those who experienced multiple types of violence (AOR = 0.38, 95% CI: 0.19, 0.79). Likewise, women who have strong social support were 2.5 times more likely to disclose their violent experience to others as compared to those who have poor social support (AOR = 2.52, 95% CI: 1.44, 4.41).

Women whose husband attain primary and secondary education were two times more likely to disclose their violent experience to others as compared to those women whose husbands attain more than secondary education (AOR = 2; 95% CI:1.07, 3.90) and (AOR = 2; 95% CI:1.07, 4.33), respectively (Table 3).

### 3.7. Qualitative Part

#### 3.7.1. Reasons for Not Disclosing Intimate Partners' Violence

Taking intimate partner violence as normal, waiting for others to let them talk about their intimate partner violence, and economic dependency on the intimate partner were the main reasons for not disclosing their experience of violence for others as most key informants were saying.

Key informant one reported, “…my mother told me that it is a part of marriage and I go back to my life and nowadays I start taking his behavior normal”.

Key informant two also reported, “One day he kicked me severely and I bleed on my left leg and went to the hospital and expecting an opportunity to be asked my experience and waiting for them to ask me about the cause and the offender but they didn't, they only treat the bleeding leg and sent me back, after that time I decided to live my life as it is with a hope that he will be changed”.

Key informant three stated, “he was the one who covered almost everything for my mother's medical fee, and school fee since I was grade 8, so how could I leave this man…all of my expenses have been covered by him”.

Fear of interference of the third party was one of the reasons for not disclosing intimate partners' violence evidenced by key informants had said.

#### 3.7.2. For Whom They Disclosed Intimate Partners' Violence

Most respondents disclosed their intimate partner violence to their family, supported by the key informants saying.

As key informants, one and three reported, “when they faced intimate partner violence they run away to their family and told their mother…”.

#### 3.7.3. Reasons for Intimate Partners' Violence

Women whose partners drink alcohol and having poor social support were the main reasons mentioned by the key informants.

Key informants one and four reported, “…he nagged me, kick me especially things worsen when he drinks alcohol and he became drunk…”.

Key informant five said, “since I have good relations with my neighbors…… I get more comfort when they told me their experience …. If they were not on my side things would become worsen”.

## 4. Discussion

This study determines the disclosure of intimate partner violence and associated factors, which is very helpful clinically for health care providers to identify the reasons behind not disclosing violence and to address it before any complication happened by screening women for intimate partner violence routinely. A total of 280 victimized women were involved in the study. The current study revealed that physical violence was the predominant next to controlling behavior and 85% of violated women were reported to be victims of multiple forms of intimate partner violence. It is much higher as compared with the study done in Southeast Nigeria that indicated 58% of violated women experienced multiple forms of intimate partner violence [12].

This study also showed 51.4% (95% CI: 44.7%-57.5%) of violated women disclosed their intimate partner violence experience to others, which is higher than the study done in Lagos, Nigeria, that found out 46% of violated women disclosed their experience to others [6]. This is also higher as compared with other studies done in Tanzania (40%) [8], Dhaka slums (21%) [26], Nigeria (28%) [6, 12], and Northwest Ethiopia (31%) [13].

The higher proportion of women disclosure in this study area might be due to the difference in gender norms and the presence of conservative cultures that support wife beating which put the women ashamed to disclose their violent experience to others that they rather accept it as part of their life; meanwhile, most of the studies were done in town, whereas this study was done in rural areas.

The majority (73%) of the women disclosed their experience of violence to their family, which is in agreement with the World Health Organization multicountry study [9] and a cross-sectional study done in Nigeria [6, 12, 24], where most women disclose their experience to their family; this might be due to fear of revenge, not wanting to get the perpetrator into trouble, the feeling that the situation was not worth reporting and to keep the situation more private [5, 24]. These outcomes further substantiate the position of the extended circle of relatives in arbitrating intimate partner conflicts, which include violence.

As this study indicates, only 8% of victim women disclosed their violent experience to health care providers that is inconsistent with a study done in Serbia, which suggests 25.7% of victim women disclosed their violent experience to health caregivers [5]. This is probably because of a lack of screening devices, cognizance, and time constraint. From the qualitative part, although individuals wanted to reveal, the findings indicate that possibilities for possible disclosure in healthcare settings are no longer an alternative because most women in the study said they have been in no way asked about intimate partner violence occurrences.

The outcomes on the motives for disclosing propose that encouragement from own family and friends and worry of the impact of the violence on children are the maximum vital triggers for a female to disclose after experiencing intimate partner violence. This is consistent with the findings coming from the WHO multicountry study which states to take a look at women and domestic violence [19]. However, women refraining from disclosing intimate partner violence to the formal institutions could also be an illustration that they lack acceptance as true with or facts on those establishments or that such institutions lack interest in domestic violence [27].

This finding is in line with a look at that accomplished in the United States of America [27] and Dhaka slums [26]. Being embarrassed, having desire to get privacy, and wanting not to contain others on personal lifestyles, fearing of revenge, taking it as a minor incident, preserving it as nonpublic, and losing belief at the police were the motives raised for no longer disclosing intimate partner violence [27]. This finding also is in keeping with research carried out in southeast Nigeria [12] and Nepal [28]. Meanwhile, encouragement from own family, fear of the impact on children, and severity of the violence are the factors recognized for the motives for disclosure of their violent experience. This is likewise in keeping with different studies performed in deferent countries like Ethiopia [13], Nigeria [24], Nepal [28], and Serbia [5].

Economical dependency turned into an additionally different aspect which hindered women from disclosing their violence to others that is supported by the quantitative result that about half of the respondents have been housewives and the maximum of the choice had been made by their husbands, so a woman may also take her home like a jail so she only does what she was told to by her husband. In such conditions, women regularly fail to disclose to others to sought assistance. This finding is in line with the different studies done in Tanzania [29] and Dhaka slum [26].

This study also showed women whose husbands attained primary and secondary education are more likely to disclose their experience as compared to those who attain more than secondary education [21, 22]. It might be because women, a wife of an educated man, are more likely to keep a secret, to keep his dignity to the level expected by society as an educated, respected, and well-conducted man.

The study also found that woman whose husband drinks alcohol is positively and significantly associated with the disclosure of intimate partner experience to others; this might be due to the fact when they consume alcohol, they may have been more likely to become aggressive and the severity of violence sustained from the drunken perpetrator and this finding is in agreement with a study done in Indonesia [15] and Colombia [25].

Women who have strong social support are more likely to disclose their violent experiences to others compared to those who got poor and moderate social support. This finding is in line with previous studies [30] with the United States of America [27, 31]; however, it is inconsistent with some studies that recognized seeking friend support can trigger negative consequences. Possible clarification is that some friends responded to disclosures and requests for help with negative reactions inclusive of judgment, disbelief, fear, anger, or blaming. These responses, in turn, can cause shame, embarrassment, fear, self-isolation, and a reluctance to disclose abuse at the part of intimate partner violence [4]. This in line with the qualitative finding which reveals fear of interference of the third party was one of the reason; this could be due to the fact that the woman become prejudgmental or the third parties were judgmental; this can display how much the society accepted male dominance in family life so that a woman in violent relation would not volunteer in disclosing her experience of intimate partner violence.

The woman reported being victimized with more than one type of intimate partner violence was more likely to disclose their violent experience to others as compared to those who were a victim of one type of violence. This may be due to the severity of the violence which is in line with a study done worldwide [32, 33].

Women may hide the information as a result of the issue being family secrecy, recall bias, and social desirability bias; not including all screened women for intimate partner violence and not getting 30 women in their home for interview were the limitations of this study.

## 5. Conclusions

Out of ten victim women, five disclosed their experience to others. Very few women disclose their condition to formal sectors such as women's affairs, health care providers, and police because of embarrassment about the condition; otherwise, the majority disclosed their condition to their family. This showed that the problem is still hidden due to family secrecy. So policymakers, gender offices, and woman's associations should develop strategies that increase women's autonomy and gender equality, involve men and educate them on the gender issue, and encourage them to engage in interspousal communication and on intimate partner violence disclosure; health care providers may help women to reduce subsequent health risks and prevent further violence by early detection of a problem which leads early management and prevents further complications.

Finally, the authors recommend for future researchers to better have men participate in the study especially on the qualitative part and to conduct further interventional research by involving all stakeholders.

## Figures and Tables

**Table 1:** Sociodemographic and behavioral characteristics of respondents (*n* = 280).

Variables	Category	Frequency	Percent
Age of women	18-24	41	14.6
	25-33	164	58.6
	34-49	75	26.8
Ethnicity	Gedeo	136	48.6
	Oromo	69	24.6
	Amhara	40	14.3
	Gurage	26	9.3
	Other (Silta, Dawuro)	9	3.2
Religion	Orthodox	87	31.1
	Protestant	123	43.9
	Muslim	42	15
	Catholic	28	10
Women's educational status	No education	28	10
	Primary education	139	49.6
	Secondary education	54	19.3
	Postsecondary	59	21.1
Partner's educational status	No education	10	3.6
	Primary education	118	42.1
	Secondary education	86	30.7
	Postsecondary	66	23.8
Women's occupational status	Housewife	88	31.4
	Daily laborer	48	17.1
	Governmental worker	65	24.9
	Merchant	38	17.4
	Nongovernmental worker	34	9.5
	Others (no job)	7	2.5
Monthly income in Ethiopian birr	<500	13	4.6
	501-1500	130	46.4
	1501-2500	101	36.1
	>2501	36	12.9
Partner alcohol use status	Yes	183	65.4
	No	97	34.6
Marital duration	<10 years	202	72.1
	>11	78	27.8

**Table 2:** Frequency distribution on types of intimate partner violence and its disclosure to whom the disclosure was made among study participants (*n* = 280).

Variable	Frequency	Percent (%)
Types of intimate partner violence (*n* = 280)	280	41.0
(1) Controlling behavior	236	84.3
(2) Physical violence	209	74.6
(3) Emotional violence	183	65.3
(4) Sexual violence	109	38.9
(5) Multiple types of intimate partner violence	239	85.5
Disclosure of intimate partner violence	144	51.4
(1) Family	107	73.8
(2) Husband's family	91	62.8
(3) Friends	76	52.8
(4) Neighbors	81	56.3
(5) Religious leader	44	30.3
(6) Health care provider	12	8.3
(7) Police	23	15.9
(8) Lawyer	8	5.5
(9) Community-based organization	17	11.9
(10) Women affair	30	20.7
(11) Local elders	40	28

**Table 3:** Bivariate and multivariate logistic regression analysis on factors associated with disclosure of intimate partner violence among victim women, in Dilla town, Ethiopia (*N* = 280).

Variable	Category	Disclosure status		COR (95% CI)	*p* value	AOR (95% CI)
		Yes	No			
Women's educational status	No education	14 (50%)	14 (50%)	1.27 (0.51, 3.12)	0.006	0.847 (0.3, 2.5)
	Primary education	74 (53.2%)	65 (46.8%)	1.44 (0.78, 2.67)		0.88 (0.4, 1.9)
	Secondary education	30 (55.6%)	24 (44.4%)	1.59 (0.75, 3.34)		1.01 (0.43, 2.34)
	Postsecondary education	26 (44.1%)	33 (55.9%)	1		1
Husband/partner educational status	No education	6 (60%)	4 (40.0%)	2.62 (0.67, 10.2)	0.046	2.1 (0.51, 8.54)
	Primary education	64 (54.2%)	54 (45.8%)	2.07 (1.12, 3.85)		2.04 (1.07, 3.9)^∗^
	Secondary education	50 (58.1%)	36 (41.9%)	2.43 (1.26, 4.70)		2.16 (1.07, 4.33)^∗^
	Postsecondary education	24 (36.4%)	42 (63.6%)	1		1
Husband alcohol use	Yes	105 (57.4%)	78 (42.6%)	2.0 (1.35, 4.06)	0.002	2 (1.18, 3.34)^∗^
	No	39 (40.2%)	58 (59.8%)	1		1
Total number. Of intimate partner violence types(IPV)	≥2 types of IPV	109 (45.6%)	130 (54.4%)	1		1
	1 type of IPV	27 (65.9%)	14 (34.1%)	0.43 (0.22, 0.87)	0.023	0.38 (0.17, 0.79)^∗^
Physical violence	Yes	103 (56.3%)	80 (43.7%)	1		1
	No	41 (42.3%)	56 (57.7%)	1.76 (1.07, 2.09)	0.075	1.58 (0.9, 2.87)
Emotional violence	Yes	114 (54.5%)	95 (45.5%)	1		1
	No	30 (42.3%)	41 (57.7%)	1.64 (0.95, 2.82)	0.026	1.35 (0.7, 2.58)
Social support	Strong	109 (55.1%)	89 (44.9%)	2.50 (1.42, 4.42)	0.001	2.52 (1.44, 4.41)^∗∗^
	Poor and moderate	27 (32.9%)	55 (67.1%)	1		1
Age at first marriage	≥18 years old	50 (45.9%)	59 (54.1%)	0.7 (0.43, 1.12)	0.225	0.95 (0.82, 1.11)
	≥18 years old	94 (55.0%)	77 (45.0%)	1		1
Age at first sexual intercourse	<18 years old	58 (45.0%)	71 (55.0%)	0.62 (0.38, 0.99)	0.046	0.62 (0.38, 1.01)
	≥18 years old	86 (57.0%)	65 (43.0%)	1		1
Able to visit family	Yes	102 (52.8%)	91 (47.2%)	1.75 (1.04, 2.92)	0.034	0.73 (0.42, 1.13)
	No	34 (39.1%)	53 (60.9%)	1		1
Contribute to the household purchase	Yes	87 (52.1%)	80 (47.9%)	1		1
	No	49 (43.4%)	64 (56.6%)	1.42 (0.88, 2.3)	0.152	1.19 (0.49, 2.91)
A decision on household purchase	Yes	79 (47.6%)	87 (52.4%)	1		1
	No	65 (57.0%)	49 (43.0%)	0.68 (0.42, 1.11)	0.122	0.82 (0.34, 1.95)

AOR: adjusted odds ratio; COR: crude odds ratio; CI: confidence interval, 1: Reference. ^∗∗^Statistically significant at *p* < 0.01; ^∗^*p* < 0.05.

## Data Availability

The data used to support the findings of this study are available from the corresponding author upon request.
